# Complete mitochondrial genome of *Gnathostoma binucleatum*

**DOI:** 10.1128/mra.00366-23

**Published:** 2024-01-24

**Authors:** Sylvia Paz Diaz-Camacho, Robert Logan, María Elena Báez-Flores, Francisco Delgado-Vargas, Rogelio Prieto-Alvarado, Inés Fernando Vega-López, Felipe Vaca-Paniagua, Clara Estela Díaz-Velásquez, Gilmar López-Armenta, Ricardo Parra-Unda

**Affiliations:** 1 Research Unit in Environment and Health, Autonomous University of Occident, Sinaloa, Mexico; 2 Eastern Nazarene College, Quincy, Massachusetts, USA; 3 Unidad de Investigaciones en Salud Pública "Dra. Kaethe Willms", Facultad de Ciencias Químico Biológicas, Universidad Autónoma de Sinaloa, Culiacán, Sinaloa, México; 4 Parque de Innovación Tecnológica, Universidad Autónoma de Sinaloa, Culiacán, Sinaloa, México; 5 Laboratorio Nacional en Salud, Diagnóstico Molecular y Efecto Ambiental en Enfermedades Crónico-Degenerativas, Facultad de Estudios Superiores Iztacala, Tlalnepantla, Estado De México, México; University of California Riverside, USA

**Keywords:** *Gnathostoma binucleatum*, mitochondrial, genome

## Abstract

This report describes the mitochondrial genome of the parasite *Gnathostoma binucleatum* (*G. binucleatum*), which was obtained from naturally infected freshwater fish in Sinaloa, Mexico (22°46′00.1″N 105°40′21.8″W). *G. binucleatum* is responsible for human gnathostomiasis and is endemic to Mexico. It belongs to the Spirurida order of the Secernentea class of Nematoda.

## ANNOUNCEMENT

*Gnathostoma binucleatum* (*G. binucleatum*) is a parasitic nematode that causes the zoonotic disease gnathostomiasis (1). Little is known about the parasite’s molecular and genetic biology, which is necessary for the development of effective anti-parasitic drugs. Only the mitochondrial MT-CO1 gene of *G. binucleatum* has been characterized (2). Here, we present the complete mitochondrial genome of *G. binucleatum* (GbMG).

Twenty-five advanced third-stage *G. binucleatum* larvae were isolated from infected freshwater fish in Tecualilla, Sinaloa, Mexico (22°46′00.1″N 105°40′21.8″W). All procedures were conducted in accordance with the International Guiding Principles for Biomedical Research Involving Animals. DNA was extracted by treating the parasites with alkaline lysis buffer (5 M NaCl, 1 M Tris pH 8, 0.5 M EDTA pH 8, 10% SDS, 40-µL proteinase K) at 55°C for 30 min, homogenizing them, and then mixing the homogenate with phenol-Tris-HCl. After centrifugation, the supernatant was transferred to a new tube, and the extraction process was repeated. The final supernatant was mixed with ethanol and centrifuged, and the pellet was then recovered and washed with 70% ethanol by pipette mixing. Following a final centrifugation, the DNA-containing ethanol supernatant was mixed with Tris-EDTA (TE) buffer and prepared for sequencing using the Illumina DNA Prep Kit.

Psomagen, Inc. (Rockville MD, USA), sequenced the samples using the Illumina MiSeq platform. A total of 44,235,384 raw reads were produced, which comprised of 22,117,692 paired-end reads (2 × 150 bp). R1 and R2 fastq files were combined and trimmed for adapters, low-quality reads, and short reads. BBDuk (Decontamination Using Kmers) version 38.84, which is part of the BBTools suite, was used for trimming (3). BBNorm version 38.84, which is also a part of the BBTool suite, was used for error correction and normalization to produce a total of 727,338 reads, 97.6% of which had a Phred score of at least Q30 (3). After pre-processing the reads, their mean length was 146 bp (SD 75 bp), with a range between 35 bp and 301 bp long. For all software tools used, the default parameters and native algorithms were chosen unless otherwise noted.

Geneious Prime version 2023.0.1 was used for *de novo* assembly, followed by mapping contigs (4). Fifteen of the *de novo* assembled contigs (with an N50 of 9.875 kb and a depth of 6) were then subsequently mapped to the mitochondrial genome reference sequence of *G. nipponicum* (NC_034239.1).

However, a consensus sequence with less ambiguity and gaps was produced when all the pre-processed 727,338 reads were mapped, resulting in a consensus sequence made from 13,163 reads.

Subsequently, Geneious performed autoannotation using the NC_034239.1 reference sequence as a gene transfer guide. To refine the draft genome, GapPredict’s machine learning algorithm filled gaps and resolved ambiguous bases, which were then confirmed manually via raw read alignment and genomic context (5). Protein-coded gene calls were confirmed to have appropriate open reading frames by using Expasy Translate with the invertebrate mitochondrial code and then queried in the non-redundant GenBank database. The top hits were from *G. nipponicum* and had high pairwise identity, affirming annotation validity. Non-protein coding genes were confirmed via blastn coordinates and *G. nipponicum* synteny. The complete annotated GbMG is 14,067 bp long, has a GC content of 28.5%, and includes 12 protein-coding genes as presented in Table 1.

**TABLE 1:** Gnathostoma binucleatum Mitochondrial Protein-coding genes

Feature	Coordinates	Anticodon	Start/stop codon	Corresponding *G.n* GeneID
COX1	1–1573		ATA/TA	31413098
tRNA-Cys	1574–1630	GCA		31413086
tRNA-Lys	1692	TTT		31413099
tRNA-Met	1708–1761	CAT		31413100
tRNA-Asp	1770–1826	GTC		31413101
tRNA-Gly	1829–1882	TCC		31413102
COX2	1883–2561		TTG/TAG	31413103
tRNA-His	2572–2627	GTG		31413087
large subunit rRNA	2624–3575			31413104
ND3	3572–3907		TTG/TAG	31413105
ND5	3913–5496		ATT/TAG	31413088
tRNA-Ala	5495–5550	TGC		31413089
tRNA-Pro	5554–5615	TGG		31413106
tRNA-Leu	5665	TAA		31413107
tRNA-Ser	5663–5720	TCT		31413108
ND2	5735–6568		ATG/TAG	31413109
tRNA-Ile	6570–6625	GAT		31413090
tRNA-Asn	7420–7475	GTT		31413110
tRNA-Arg	7508–7564	TCG		31413111
tRNA-Gln	7563–7617	TTG		31413112
tRNA-Phe	7618–7687	GAA		31413113
CYTB	7713–8782		ATT/TAG	31413114
tRNA-Leu	8785–8839	TAG		31413091
COX3	8840–9607		TTG/TAG	31413115
tRNA-Thr	9609–9668	TGT		31413092
ND4	9684–10895		TTG/TAA	31413116
tRNA-Tyr	10896–10946	GTA		31413093
ND1	10947–11822		TTG/TAA	31413117
ATP6	11908–12418		ATT/TAG	31413094
tRNA-Val	12468–12521	TAC		31413095
ND6	12522–12959		ATA/TAG	31413118
ND4L	12967–13193		TTG/TAG	31413096
tRNA-Trp	13194–13248	TCA		31413097
tRNA-Glu	13253–13306	TTC		31413119
Small subunit rRNA	13309–13979			31413120
tRNA-Ser	13981–14034	TGA		31413121

## Data Availability

This sequence has been deposited in GenBank under the accession number OQ842461.1, and the raw data have been deposited in SRA with the accession number PRJNA1030759.
